# Adolescent Self-Organization Predicts Midlife Memory in a Prospective Birth Cohort Study

**DOI:** 10.1037/a0033787

**Published:** 2013-12

**Authors:** Man K. Xu, Peter B. Jones, Jennifer H. Barnett, Darya Gaysina, Diana Kuh, Tim J. Croudace, Marcus Richards

**Affiliations:** 1 Department of Psychiatry, University of Cambridge, Cambridge, United Kingdom; 2 Department of Psychiatry, University of Cambridge and Cambridge Cognition, Cambridge, United Kingdom; 3 School of Psychology, University of Leicester, Leicester, United Kingdom; 4 MRC Unit for Lifelong Health and Ageing, London, United Kingdom; 5 Department of Psychiatry, University of Cambridge and Department of Health Sciences and Hull York Medical School, University of York, York, United Kingdom; 6 MRC Unit for Lifelong Health and Ageing and MRC National Survey of Health and Development scientific and data collection team, London, United Kingdom

**Keywords:** self-organization, memory, life course

## Abstract

Childhood and adolescent mental health have a lasting impact on adult life chances, with strong implications for subsequent health, including cognitive aging. Using the British 1946 birth cohort, the authors tested associations between adolescent conduct problems, emotional problems and aspects of self-organization, and verbal memory at 43 years and rate of decline in verbal memory from 43 to 60–64 years. After controlling for childhood intelligence, adolescent self-organization was positively associated with verbal memory at 43 years, mainly through educational attainment, although not with rate of memory decline. Associations between adolescent conduct and emotional problems and future memory were of negligible magnitude. It has been suggested that interventions to improve self-organization may save a wide range of societal costs; this study also suggests that this might also benefit cognitive function in later life.

Mental health problems occurring in childhood have a profound impact on life chances. This is particularly true of conduct problems, which are associated with poor educational and socioeconomic attainment, weak family ties, increased likelihood of trouble with the criminal justice system, risky health-related behaviors, poor physical health, increased adult psychiatric disorder, and premature mortality (Colman et al., 2009; Fergusson, Horwood, & Ridder, 2005; Jokela, Ferrie, & Kivimäki, 2009; Moffitt et al., 2011; Richards & Abbott, 2009). Although such outcomes are less marked for childhood emotional problems, the latter are nevertheless associated with lower levels of social participation and affiliation, including partnership (Hatch & Wadsworth, 2008; Richards & Abbott, 2009) and also increased risk of psychiatric disorder (e.g., Colman, Wadsworth, Croudace, & Jones, 2007; Fergusson et al., 2005; Weissman et al., 1999). Because many of the above outcomes, particularly poor educational attainment (Albert et al., 1995), risky health behaviors (Leventhal, Rabin, Leventhal, & Burns, 2001), and poor adult physical (Weuve et al., 2004) and mental health (Starkstein, Mayberg, Leiguarda, Preziosi, & Robinson, 1992) have an adverse impact on cognitive aging, it would not be surprising if childhood mental health problems also lead, over the longer term, to increased risk of dementia. This is an important issue, in view of the huge individual and societal costs of Alzheimer’s disease (Richards & Brayne, 2010), the most common of the late-life dementias. However, although conduct problems in early childhood were found to be associated with poorer subsequent childhood cognition in population-based studies such as the British, 1970 birth cohort (Richards & Abbott, 2009), we are not aware that any studies have traced the long-term consequences of this for cognitive aging and decline. Similarly, although several studies suggest that adult psychiatric disorder is negatively associated with cognitive aging (Starkstein et al., 1992), few if any studies to date have investigated the longer term impact of preadult mental problems on this aging outcome.

The 1946 British birth cohort study, one of the longest continuously running studies of human development and aging in the world, offers an excellent opportunity to test associations between adolescent mental health problems and future memory, a key cognitive predictor of dementia (DeCarli et al., 2004). School teachers were asked to rate the behavior of study members when they were aged 13 and 15 years, according to 28 different criteria. Memory was first measured in this cohort at age 43 years, then again at 53 and 60–64 years, thus, allowing investigation of rate of future memory decline. We hypothesized that study members with adolescent conduct problems would show poorer memory at 43 years and faster memory decline, than those without these problems. Because conduct problems are associated with poor educational attainment (Richards & Abbott, 2009), which in turn is associated with lower adult cognitive performance (Clouston et al., 2012) and, in some studies, with faster cognitive decline (Anstey & Christensen, 2000), we also hypothesized that associations between adolescent conduct problems and poor future memory would be significantly mediated through low educational attainment. In addition, a special advantage of the 1946 birth cohort is that cognition was assessed in childhood, several years prior to the teacher ratings of adolescent mental health. This enabled us to control for possible reverse causality, that is, that any apparent associations between conduct problems and future memory were actually driven by poor cognitive development, which in turn tracks across the life course and, thus, leads to poor future memory performance (Richards & Sacker, 2003) and, in some studies, faster memory decline (Gow et al., 2012; Richards, Shipley, Fuhrer, & Wadsworth, 2004).

Studies of adolescent mental health based on behavioral data rated by parents and teachers have largely focused on dimensions representing conduct and emotional problems; however, not all aspects of mental health neatly fit into these two relatively independent dimensions. In particular, there is a growing interest in the role of adolescent self-control (Moffitt et al., 2011) and self-organization (K. Duckworth, Akerman, MacGregor, Salter, & Vorhaus, 2009), the poor development of which are likely to be closely related to conduct problems. For example, although disobedience and attention seeking clearly belong to conduct problems, laziness and lack of diligence in the classroom may more accurately reflect poor self-organization and control. Using the Dunedin Multidisciplinary Health and Development Study, Moffitt et al. (2011) found that lower childhood self-control predicted poorer socioeconomic status, lower income, poorer financial planning, more frequent financial struggles, poorer physical health, greater substance dependency, greater likelihood of single-parent child rearing, and greater likelihood of a criminal court conviction. As with conduct and emotional problems, it is reasonable to anticipate that poor self-organization and control will also lead to future memory problems through lack of self-care in regard to health. However, this may also arise through more direct biological mechanisms. Indeed, self-organization has been defined as “self-generated thoughts, feelings and actions that are planned and cyclically adapted to the attainment of personal goals” (Zimmerman, 2000, p. 14); this echoes accepted definitions of executive function, an important component of many memory tasks. As part of this study we, therefore, revisited the adolescent behavior ratings to investigate whether self-organization could be identified as a dimension separate from those of conduct and emotional problems; and, if so, to test the relative influence of all three mental health factors on future memory.

## Method

### Sample

The Medical Research Council National Survey of Health and Development (NSHD), also known as the British 1946 birth cohort, originally consisted of a socially stratified sample of 5,362 singleton children (girls: *n* = 2547, 47.5%; boys: *n* = 2815, 52.5%) born within marriage in one week in March 1946 in mainland Britain, with regular follow-up across life (Kuh et al., 2011). The most recent follow-up was between 2006 and 2010 (at 60–64 years, henceforth 60+ years). In total 2856 eligible study members (those known to be alive and with a known address in England, Scotland, or Wales) were invited for an assessment at one of six clinical research facilities (CRFs) or to be visited by a research nurse at home. Invitations were not sent to those who were already known to have died (*n* = 778), who were living abroad (*n* = 570), had previously withdrawn from the study (*n* = 594), or had been lost to follow-up (*n* = 564). Of those invited, 2,229 (78%) were assessed: 1,690 (59.2%) attended a CRF and the remaining 539 were seen at home (Stafford et al., 2013). The current study protocol received ethical approval from the Greater Manchester Local Research Ethics Committee for the four English sites; the Scotland A Research Ethics Committee approved the data collection taking place in Edinburgh. Written informed consent was obtained from the study member at each stage of data collection.

### Adult Memory Test

At either the clinic or home visit, study members undertook the same tests of verbal memory and timed letter search given at ages 43, 53 (Richards et al., 2004), and 60+ years. This consisted of a 15-item word-learning task devised by the NSHD. Each word was shown for 2 s. When all 15 words were shown, the study member was asked to write down as many of these as possible, in any order. Three repeated trials were conducted on each testing occasion. In this study, the sum of scores of correctly remembered items at each test trial from each assessment was used as an indicator for a latent variable construct representing memory performance. The distributions of these nine test items of verbal memory approximated a normal distribution in terms of minimal skewness and kurtosis. The mean skewness value was −0.11 (the skewness for a standard normal distribution is zero), with no item larger than 0.32 or lower than −0.50. The mean kurtosis value was 2.96 (the kurtosis for a standard normal distribution is 3), with no item larger than 3.10 or lower than 2.77.

Two different lists of words were applied to the memory assessment in an effort to avoid the impact of “practice” effects. At the first assessment (age 43 years), study members randomly received one of two different word lists, which were then alternated over the subsequent two data collections. To adjust for any potential bias introduced by word list, a binary variable representing test type was included in the analysis as a covariate. Because the most recent assessment took place between ages 60 and 64 years, age at assessment was incorporated into the model estimation to account for any differences in memory that might result from this. This was integrated into the latent growth curve model through the TSCORE command within Mplus 6.1 (Muthén & Muthén, 2010) latent variable modeling software. The TSCORE function takes into account the individually varying times of observation resulting from the fieldwork data collection. Both the baseline measure and the slope representing memory decline were, in return, specified in an overarching structural equation model as outcomes in relation to childhood cognition, adolescent mental health, and educational attainment.

### Adolescent Mental Health

Teachers were asked to rate study members on a three category response scale where they had to compare the study member’s behavior with that of “a normal child” at ages 13 and 15 years, using items that were forerunners of those used in the Rutter A scale (Elander & Rutter, 1996; Rutter, Tizard, & Whitmore, 1970). Previous work has used factor analysis of these ratings to identify two dimensions, emotional and conduct problems, under traditional (Rodgers, 1990) and categorical (Colman et al., 2009) data factor analysis.

For our study, teacher rating data at age 13 and 15 years were resubjected to separate exploratory factor analysis at these ages. Item level data were modeled as ordinal using probit models for categorical outcomes in the statistical package Mplus 6.1 (Muthén & Muthén, 2010). Some items clearly measured two different dimensions of mental health; for example, for the item “How does this child react to criticism or punishment?” teachers were asked to choose from *unduly resentful*, *normal attitude to criticism and punishment*, and *tends to become unduly miserable or worried*. To integrate such items into the factor analysis appropriately, we recoded these responses into two binary items, each representing a new variable compared with a normal child. These recoded binary items loaded substantially and uniquely on corresponding factors. Table 1 shows a list of the items recoded in this way, along with their factor loadings.[Table-anchor tbl1]

Examination of scree plots, eigenvalues, and model fit indices suggested a three-factor solution for this set of items, each representing emotional problems (e.g., gloomy and sad, extremely fearful), conduct problems (e.g., disobedience, evading truth to keep out of trouble), and self-organization. The self-organization factor was defined by items relating to attitude to work; concentration; neatness in work; and not daydreaming in class (see the Results section for details). Factor scores at ages 13 and 15 years were summed to create scales representing these dimensions, and to facilitate the interpretation, the new combined scales were then standardized to form *z* scores.

### Childhood Cognition

Childhood cognitive ability at age 8 years was represented as the sum of four tests of verbal and nonverbal ability devised by the National Foundation for Educational Research (Pigeon, 1964). These tests were (a) Reading comprehension (selecting appropriate words to complete 35 sentences); (b) Word Reading (ability to read and pronounce 50 words); (c) Vocabulary (ability to explain the meaning of 50 words); and (d) Picture Intelligence, consisting of a 60-item nonverbal reasoning test. We used confirmatory factor analysis to construct a scale summarizing these data. Model fit indices (see later description on fit indices) were: chi-square = 63.145 with 1 *df*, root-mean-square error of approximation (RMSEA) = .121, comparative fit index (CFI) = .994, Tucker-Lewis index (TLI) = .966. Factor scores were computed then standardized to a mean of zero with a standard deviation of one.

### Educational Attainment

The highest level of educational qualification attained by age 26 years was coded by the U.K. Burnham scale (Department of Education and Science, 1972) and recoded for the present analysis into no qualification (*n* = 1,765, 39.8%); vocational only (*n* = 353, 8.0%); “O level” (secondary level taken by public examination at age 15 years, *n* = 863, 19.5%); “A level” (advanced secondary level qualifications taken by public examination at 18 years, *n* = 1,040, 23.5%); and tertiary level (degree or equivalent, or higher degree, *n* = 411, 9.3%). Similar to adolescent mental health and childhood cognition, the educational attainment variable was standardized to a mean of zero with standard deviation of one.

### Statistical Analysis

We applied structural equation models (Skrondal & Rabe-Hesketh, 2004; Tabachnick & Fidell, 2006) incorporating latent growth analyses (Ferrer, Balluerka, & Widaman, 2008; Hancock, Kuo, & Lawrence, 2001) to describe the way that childhood cognition, the three adolescent mental health factors, and educational attainment were associated with verbal memory at 43 years (intercept) and rate of decline in verbal memory from 43 to 60+ years (slope). This model contained two main components: (a) paths from childhood cognition to the three adolescent mental health factors and to education; (b) simultaneous paths from these variables to memory baseline and rate of decline. We first examined the measurement properties of the memory construct over time. The goodness of fit of these models was evaluated by a range of recommended indices including the TLI (Tucker & Lewis, 1973), the RMSEA (Steiger, 1990), and the CFI (Bentler, 1990). Comparative fit indexes and TLIs greater than .95 are often taken to indicate an acceptable model fit, whereas the RMSEAs less than .06 indicate good fit. We also present the chi-square statistic; however, this is highly sensitive to large sample sizes (Browne & Cudeck, 1993), making it a less suitable index for this study.

#### Measurement invariance of the latent memory variables

Because memory at three different ages (43, 53, and 60+ years) provided the basis of age-related change, it is important to assess the extent to which the memory tests were comparable across these three sets of assessments. Thus, we conducted measurement invariance tests prior to fitting a latent growth model of memory. This was done through assessing model fit of a set of increasingly restrictive models. The model fit indices were compared to evaluate the degree of invariance of the measurement parameters in the models. The baseline model tested configural invariance, whereas the latent memory variable had the same number of factor indicators, that is, three trials at each occasion. In this model, the factor loadings were set to be freely estimated across the assessments. This model was a prerequisite for testing the next step, the metric invariance, where the factor loadings were constrained to be equal across assessments. This step ensured that the memory construct had the same substantive meaning across assessments. In the last step, scalar invariance was assessed by additionally constraining the intercepts of the indicators to be equal across assessments. This step validated the comparison of the latent means of latent memory variables across assessments, which was essential for fitting a latent growth curve model. We used the same range of fit indices to investigate models of measurement invariance. A restrictive model is preferred if the change in model fit indices are not significantly inferior to those of the less restrictive model. In terms of the RMSEA, the change should be less than .015 (Chen, 2007). For CFI, the change should be less than .01 (Chen, 2007; Cheung & Rensvold, 2001). We also controlled for the effect of test type with a multiple-indicator-multiple-cause (MIMIC) modeling approach (Kaplan, 2000). Regression paths were specified between test type variable and latent memory variables at each assessment. Paths from test type to each of the memory test trial indicators were first constrained to be zero, assuming no effect from test type to memory indicators. Then modification indices were examined to evaluate whether this assumption was true. Paths associated with high modification indices were subsequently freed to control for any measurement bias in the latent memory variable items introduced by test type. Covariances of residual variances of test trials across assessments were estimated a priori, as it is expected that tests repeatedly assessed at different time points are auto-correlated.

#### Model estimation and mediation effects of adolescent mental health

All factor analyses and latent variable structural equation models were estimated using Mplus 6.1. The estimator used for the measurement invariance tests was maximum likelihood. The model estimator for the overall SEM was maximum likelihood with robust standard errors. In this study, educational attainment was a hypothesized mediator between adolescent mental health and memory decline. It is not possible in MPlus to request model output on the mediation effect when the robust maximum likelihood estimator is used together with TYPE = RANDOM and TSCORE. Hence, we calculated the mediation effect of adolescent mental health using the MODEL CONSTRAINT function in Mplus 6.1, following the mediation formula for continuous variables (Hayes, 2009; von Soest & Hagtvet, 2011).

## Results

### Missing Data

Study members with missing adult data had poorer cognition at age 8 years, more adolescent emotional and conduct problems, lower self-organization, lower educational attainment, and lower midlife memory scores. There was a general, slight trend of memory decline from 43 to 60+ years. Means and standard deviations (in parenthesis) for the overall memory score at 43, 53, and 60+ years, when only those with complete cognitive data were included (*n* = 1,875): 25.55 (6.09), 24.78 (6.05), and 24.37 (6.09), respectively.

### Adolescent Mental Health Symptoms

Exploratory factor analysis suggested three dimensions within the teacher ratings (see Method section), representing emotional problems (e.g., gloomy and sad, extremely fearful), conduct problems (e.g., disobedience, evading truth to keep out of trouble), and the new factor characterized as self-organization. The details of this analysis are presented in Table 1. The putative self-organization factor was defined by four items relating to attitude to work; level of concentration; degree of neatness in work; and extent of daydreaming in class (see Table 1). For ease of interpretation, this factor was coded so that higher scores indicated better self-organization, whereas following convention higher scores for the emotional and conduct factors indicated more severe problems.

### Longitudinal Measurement Invariance of Verbal Memory

We fitted a series of CFA models assessing the measurement invariance of latent memory variables as described above (see Table 2). Model m0 represented the configural invariance model, where all measurement parameters were freely estimated. Model m1 was the metric invariance model where factor loadings of memory latent variable were constrained to be equal across the three occasions of memory measurement. Model m2 was the scalar invariance model, for which, in addition to factor loadings, item intercepts were also constrained to be equal across assessment occasions. This was the most strict model and represented full measurement invariance necessary for the present investigation. Models m0, m1, and m2 all showed excellent fit indices, and little change was observed in the fit indices despite increasing model restriction, thus, indicating good measurement invariance of latent memory variables across time (see Table 2). Thus, it was valid to compare the latent memory variables across time, which provided a basis for fitting the latent growth curve models.[Table-anchor tbl2]

To investigate the effect of test type (the two different word lists in the memory test), in Model m3, we first constrained regression paths from test type to be 0 to each of the test-trial indicators (see Table 2). Modification indices were then examined to identify test trial indicators with high modification indices. In Model m4, paths associated with these indicators were allowed to be freely estimated. Model m4 represented the final measurement model specification for memory in all subsequent analysis. We fitted a CFA model incorporating all variables included in the present investigation. This model had good fit indices, demonstrating a close fit to the data (RMSEA = .017, CFI = .994, TLI = .99). Zero-order correlations of these variables are shown in Table 3.[Table-anchor tbl3]

### Cognitive Decline and Its Predictors

**Figure 1 fig1:**
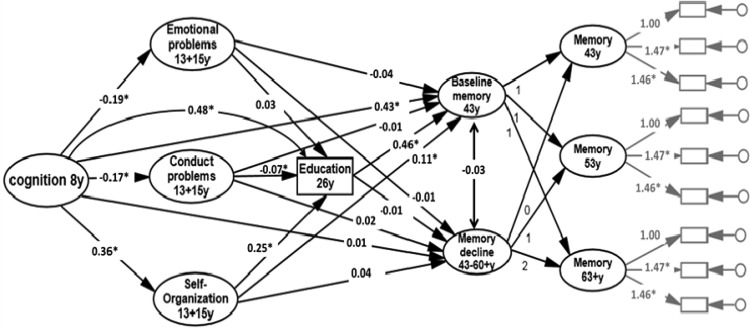
Longitudinal path diagram representing associations between childhood cognition, adolescent mental health, educational attainment, and memory at 43 years (baseline) and from 43 to 60–64 years (rate of decline). * *p* < .01. Measurement invariance of the specific memory tests at each occasion were assessed (see Table 2). For ease of reading, paths from gender are omitted from the figure. The rectangular boxes on the right in gray represent the three memory task trials at each age.

Figure 1 represents the results of an overarching structural equation model that shows paths linking childhood cognition, the above three adolescent mental health dimensions, educational attainment, and future memory baseline (at 43 years) and memory decline (from 43–60+ years). Paths (see Table 4) are adjusted for gender and are all statistically independent of each other. The path values are standardized regression weights, so should be interpreted in metrics of standard deviations of the predictor and outcome variables.[Fig-anchor fig1][Table-anchor tbl4]

The intercept of the second-order latent growth curve model represented the baseline memory function at age 43 years. Latent mean of the baseline was set to zero for the estimation of latent means at the later ages 53 years and age 60+ years. The mean and significance level of the growth factor slope parameter (Part B, Table 4) were −0.10, *p* = .004, indicating a moderate decline in verbal memory function. No variable in the model, including self-organization, was significantly associated with rate of memory decline. The residual variance of the slope parameter was statistically nonsignificant (*p* = .48), indicating a lack of individual differences in the slope.

It can be seen that childhood cognition was inversely associated with conduct and emotional problems and positively associated with self-organization, and it was positively associated with verbal memory at age 43 (Table 4, Part B). However, net of this influence, adolescent self-organization was positively associated with memory at 43 years. This was mainly mediated by educational attainment (see mediation results in Table 5), although a direct path from self-organization to future memory can also be observed (the direct effect was 0.11, the indirect effect was 0.11, and the total effect was 0.23). In contrast, independent inverse associations between adolescent conduct and emotional problems and future memory were of negligible magnitude. However, conduct problems had a small indirect effect on memory baseline at age 43 through education (see Table 4). Childhood cognition was directly and positively associated with educational attainment and with memory at 43 years. Educational attainment was positively associated with memory at 43 years. This was not only independent of prior cognition but was also independent of adolescent mental health.[Table-anchor tbl5]

## Discussion

In this longitudinal prospective population-based study, we tested associations between dimensions of adolescent conduct, emotional and self-organization problems derived from teacher ratings, level of future verbal memory, and rate of memory decline, from early to late middle age (43 to 60–64 years). This approach also allowed us to assess whether any such associations were independent of prior cognition in childhood and assess the extent to which they were mediated by educational attainment. None of the adolescent mental health factors were associated with rate of memory decline, and neither emotional nor conduct problems were directly associated with memory performance at 43 years. However, self-organization was strongly, positively, and directly associated with better memory performance when gender, childhood cognition and educational attainment were controlled. Self-organization was also indirectly associated with memory at age 43 through educational attainment. Adolescent conduct problems had an indirect inverse effect, although of smaller magnitude, but this was not the case for emotional problems. As expected from previous analyses in NSHD (Richards & Sacker, 2003), there were strong direct associations between childhood cognition and educational attainment and memory at age 43 years, although neither childhood cognition nor educational attainment were associated with rate of memory decline, contrary to previous studies using regression analysis based on observed change scores (Gow et al., 2012). However, our analysis, based on latent variable modeling, circumvented some of the potential biases related to missing data and measurement error, thus, representing a more valid approach compared with methods based only on manifest variables (Christensen et al., 2001).

Our study has strengths and limitations. Regarding the latter, individuals with poorer adolescent mental health, lower cognitive ability, and lower educational attainment were underrepresented, as in many longitudinal studies. However, the longitudinal nature of NSHD, and our use of structural equation modeling allowed us to use full information maximum likelihood estimation (Graham, 2009), thus, accounting for missing data that could result in a biased sample if list-wise deletion was used. There were also relatively few items in the teacher rating scale that identified self-organization. However, as noted in a commentary (A. L. Duckworth, 2011) to the Moffitt et al. (2011) study, the latter authors use the related term *self-control* synonymously with *conscientiousness*, which involves industriousness and orderliness, two of the four items belonging to self-organization in our study. Furthermore, this factor was shown to predict our study outcomes differentially from the other adolescent factors. Our study also has major strengths: the national population-based sample; the availability in the same study members of directly assessed cognitive ability in childhood and future memory function in midlife; independently and prospectively rated adolescent mental health; and the repeated measures of verbal memory over a 20-year period from early to late midlife.

Because conduct problems have a striking negative impact on educational and occupational attainment and on risky health-related behaviors (Colman et al., 2009; Fergusson et al., 2005; Moffitt et al., 2011; Richards & Abbott, 2009), we expected to observe negative consequences of this for midlife memory. However, once the new factor of adolescent self-organization was identified, which included an item previously identified as belonging to the conduct factor (daydreaming in class, 3), it was this factor that showed the strongest association with future memory, with conduct problems only having an indirect (and modest) effect through educational attainment (see Table 4). As noted, self-organization also had an additional effect on future memory through educational attainment, which is consistent with earlier work in NSHD showing that education has a positive effect on future memory independently of social class of origin, childhood cognition, and adult social class (Hatch, Feinstein, Link, Wadsworth, & Richards, 2007; Richards & Sacker, 2003). It is also worth noting here that, in an earlier NSHD study, Kuh, Head, Hardy, and Wadsworth, (1997) showed that females rated by a teacher as being a “very hard worker” in adolescence subsequently had higher adult earnings, mediated by educational attainment. This factor of self-organization therefore deserves further consideration.

In essence, self-organization involves actions and strategies for attaining goals and for devising and enacting alternatives when these goals are blocked or the original actions and strategies are ineffective (Lerner et al., 2011). It is a “core aspect of human functioning” (Geldhof & Little, 2011, p. 46) that shapes development through transitions from childhood to adulthood and across life (Geldhof & Little, 2011; Maniar & Zaff, 2011). It is synonymous with self-control, which Moffitt et al. (2011, p. 2693) referred to as an “umbrella construct that bridges concepts and measurements from different disciplines (e.g., impulsivity, conscientiousness, self-regulation, delay of gratification, inattention-hyperactivity…).” Given the four items that positively load the self-organization factor in NSHD (very hard worker; high power of concentration; extremely neat and tidy in class; seldom or never daydreams in class), it is not difficult to imagine why these are highly predictive of educational attainment. Indeed, McClelland and Cameron (2011) noted that ability to direct attention is important for school success, even in children who are emotionally reactive; and self-organization also provides a foundation for positive classroom behavior through good relations with peers and teachers. This is in line with self-determination theory, which emphasizes the fundamental human goals of autonomy, competence, and relatedness (e.g., Deci & Ryan, 1991).

Education, in turn, had a positive influence on memory, not only after taking account of selection into educational attainment by prior (childhood) cognition, as previously shown by Richards and Sacker (2003), but also by adolescent behavioral characteristics, including self-organization. In view of the recent debate over whether education has a causal effect on subsequent cognition (Richards & Sacker, 2011), this reinforces the suggestion that factors intrinsic to the educational process, in addition to characteristics that pupils bring to the classroom, have a lasting impact on cognitive function. Schooling teaches specific knowledge, teaches practical skills for the workplace, refines other cognitive skills, and socializes the individual for success, all of which are likely to enhance self-organization further through a recursive process (see Richards & Hatch, 2011). Educational achievement then optimizes a variety of channels to health through economic resources and better self-care (Ross & Wu, 1995). In the specific context of memory, there is also evidence that formal schooling has a causal influence on memory through exposure to declarative (facts) and procedural (rules) knowledge and through the way in which material to be recalled is organized (Ceci, 1991), again an executive process.

However, adolescent self-organization was also associated with midlife memory in our study, independently of education. This may be partly because of factors not incorporated into our model, such as health-related behaviors, although these are themselves strongly patterned by education (Mirowsky & Ross, 1998). In this context, poor self-control was associated with substance problems in the Dunedin study (Moffitt et al., 2011); and smoking, for example, was associated with poorer future memory in NSHD (Richards, Jarvis, Thompson, & Wadsworth, 2003). However, it is also possible that the association between self-organization and future memory that is independent of education may partly reflect a direct biological pathway. This could be a process of genetic common cause (Thapar & Rutter, 2009), although it should be noted that Moffitt et al. (Moffitt et al., 2011) found that self-control was associated with a wide range of outcomes even in a substudy using a between-sibling design. Alternatively, the possibility of a direct biological path may be understood in the context of frontally regulated executive function. Executive function is a complex cognitive construct, involving diverse mental operations such as response inhibition, set-shifting and spatial and temporal sequencing. However, executive tasks also often involve retrieval, as required for example in verbal fluency tasks such as animal naming; and what Hofmann, Schmeichel, and Baddeley (2012) referred to as “updating” of relevant information, which closely connects with working memory, defined by Hofmann et al. as “the ability to keep information in an active, quickly retrievable state, and shield this information from distraction” (p. 174). Accordingly, a connection between self-organization and working memory may arise because the former involves, among other processes, top-down control of attention toward goal-relevant information and away from attention-grabbing material, ruminative thoughts, and competing affect (Hofmann et al., 2012). However, in a recent neuroimaging study, a more efficient neural network use apparently involved in an association between higher self-control in early childhood and more controlled working memory in midlife did not include frontal regions (Berman et al., 2013). More evidence is needed to clarify this issue.

We should note that down-regulation of affect may explain the null association between emotional problems and future memory in NSHD, when a negative association might have been expected. It is worth noting, however, that, this null finding may be because the cohort was not yet old enough for memory decline to be sufficiently pronounced, or variable between individuals. Hence it will be important to revisit this when study members are at a higher age risk for clinically significant cognitive decline.

In conclusion, to our knowledge this is the first prospective investigation of the association between adolescent mental health and cognitive aging, taking into account potential selection bias by prior cognitive ability and effect mediation through education. We anticipated that adolescent conduct problems would have a negative association with midlife memory, since these can have a devastating impact on life chances, leading to social disadvantage and poor health. This expectation was not confirmed. However, the striking finding was the positive association between adolescent self-organization and future memory, which was partly mediated by educational attainment, but was also a direct effect with a plausible biological basis. The factor of self-organization correlates inversely with that of conduct problems, but nevertheless remains a distinct entity in our model. If in the longer term low-adolescent self-organization is found to be a significant predictor of dementia incidence, then this suggests an important target for early preventative intervention. Indeed the promotion of self-regulation is already a government/NGO goal, as exemplified, for example, by the UNESCO International Bureau of Education “Tools of the Mind” strategy (Bodrova & Leong, 2007); and the U.K. government document “The Children’s Plan” (U.K. Department for Education, 2007). Although early evaluation of the former is not encouraging (Barnett et al., 2008), our study reinforces the long-term need for further evaluation of this prevention-oriented approach.

## Figures and Tables

**Table 1 tbl1:** Factor Loadings and Descriptions of the Teacher-Rated Items

Age 13 EFA loading			Age 15 EFA loading				Item descriptions
Org	Con	Emo	Org	Con	Emo		
.82	.32	−.02	.93	.05	−.15	1	Which statement in each group best describes this child? A very hard worker 1; average–works moderately well 2; a poor worker or lazy 3
.74	.35	.06	.92	−.01	−.05	2	One with high power of concentration 1; Average–concentrates moderately well 2; little or no power of sustained concentration 3
.47	.33	−.03	.58	.05	−.11	3	Extremely neat and tidy in class work 1; average–moderately neat and tidy 2; very untidy in class work 3
.41	.27	.31	.59	.03	.19	4	Seldom or never daydreams in class 1; sometimes daydreams in class 2; frequently daydreams in class 3
.23	.47	.10	.39	.34	.02	5	Has this child been punctual in attending school during the past year? Never late unless with good reason 0; Sometimes late 1; Persistently late 2
.20	.43	.23	.43	.38	.03	6	Has this child played truant during the last year? Yes, frequently 2; yes, occasionally 1; Never 0
.08	.85	−.16	.09	.83	−.13	7	Seldom or never disobedient 1; Sometimes disobedient 2; Frequently disobedient 3
−.01	.91	−.10	.04	.90	−.07	8	Seldom or never difficult to discipline 1; sometimes difficult to discipline 2; frequently difficult to discipline 3
.23	.74	−.15	.35	.52	−.21	9	Seldom or never restless in class 1; Sometimes restless in class 2; Frequently restless in class 3
.27	.56	.12	.44	.34	.01	10	Seldom or never cribs 1; Sometimes cribs 2; Frequently cribs 3
.11	.73	.18	.27	.63	.13	11	Seldom or never evades the truth to keep out of trouble 1; Sometimes evades the truth to keep out of trouble 2; Frequently evades the truth to keep out of trouble 3
.05	.67	−.11	.00	.67	−.21	12^a^	Liable to get unduly rough during playtime 1; Takes a normal part in rough games 2
−.02	.77	−.15	.02	.72	−.25	13^a^	Does not unduly avoid or seek attention 2; Shows off; seeks attention 3
.01	.63	−.55	.03	.52	−.57	14^a^	A dare devil 2; As cautious as the average child 1
−.10	.88	.16	−.25	1.01	.08	15^a^	A quarrelsome and aggressive child 1; Average–not particularly quarrelsome 2
−.46	.71	−.01	−.59	.89	−.03	16^a^	Overcompetitive with other children 1; normally competitive 2
−.17	.80	.22	−.13	.86	.16	17^a^	How does this child react to criticism or punishment? Tends to become unduly resentful 1; Normal attitude to criticism and punishment 0
.30	−.08	.59	.32	−.05	.56	18	Do you regard this child as Extremely energetic, never tired 1; Normally energetic 2; Always tired and “washed out” 3
−.03	−.17	.79	.05	−.17	.74	19^a^	Takes a normal part in rough games 2; Rather frightened of rough games 3
.06	−.22	.61	.04	−.17	.67	20^a^	Avoids attention, hates being in the limelight 1; Does not unduly avoid or seek attention 2
.14	−.02	.85	.05	.14	.88	21^a^	As cautious as the average child 1; Extremely fearful 3
.01	.29	.48	.01	.44	.51	22	Unusually happy and contented child 1; Generally cheerful and in good humor 2; usually gloomy and sad 3
.08	−.34	.81	.10	−.29	.83	23^a^	Average—not particularly quarrelsome 2; A timid child 3
−.03	.09	.61	−.08	.15	.62	24	Makes friends extremely easily 1; Takes usual amount of time to make friends 2; does not seem able to make friends 3
.34	.01	.67	.34	.00	.62	26^a^	Normally competitive 2; diffident about competing with other children 3
−.17	.02	.67	−.15	.12	.72	27	Would you describe this child as an anxious child (i.e., apprehensive, worrying, and fearful)? Not at all anxious 0; somewhat anxious 1; very anxious 2
−.23	.24	.70	−.18	.28	.69	28^a^	How does this child react to criticism or punishment? Tends to become unduly miserable or worried 2; Normal attitude to criticism and punishment 0
*Note*. Org = self-organization; Con = conduct problems; Emo = emotional problems. For age 13, model fit indices were χ^2^(273) = 1229.862, root-mean-square error of approximation (RMSEA) = .029, comparative fit index (CFI) = .977, Tucker-Lewis index (TLI) = .971. For age 15, model fit indices were χ^2^(273) = 1431.080, RMSEA = .032, CFI = .976, TLI = .969.							
^a^ This item originally was presented as part of a single question but was subsequently recoded into two separate binary items to represent better the meaning of the question (see the Method section for more detail). Self-organization is coded as higher score = better organization.							

**Table 2 tbl2:** Model Fit Indices of Measurement Invariance Models

Model	χ^2^	*df*	RMSEA	CFI	TLI	Base	Δχ^2^	Δ*df*	ΔRMSEA	ΔCFI	ΔTLI	Longitudinal invariance
Longitudinal measurement invariance of cognition indicators												
m0	147.322	15	.051	.992	.981							inv = none, free = fl + inter + res
m1	171.692	19	.049	.991	.983	m0	24.37	4	−.002	−.001	.002	inv = fl, free = inter + res
m2	312.74	25	.058	.983	.975	m1	141.048	6	.009	−.008	−.008	inv = fl + inter, free = res
Measurement invariance (item intercept) in relation test type												
m3	339.017	35	.052	.981	.976							regression paths from test type to item intercepts fixed to 0
m4	248.328	30	.048	.987	.980	m5	−90.689	5	−.004	.006	.004	regression paths from test type to item intercepts freed for 5 indicators
*Note*. CFI = comparative fit index; TLI = Tucker-Lewis index; RMSEA = root-mean-square error of approximation; inv = parameters constrained to be invariant across the multiple groups; fl = factor loadings; res = item residual variances; Inter = item intercepts.												

**Table 3 tbl3:** Correlation Matrix of Variables Included in the Latent Growth Structural Equation Model Path Analysis

Variable	1	2	3	4	5	6	7	8	9
1. Sex	—								
2. Cognition age 8 years	.00	—							
3. Educational attainment	−.13	.56	—						
4. Self-organization age 13 + 15 years^a^	.17	.36	.43	—					
5. Conduct problems age 13 + 15 years	−.13	−.16	−.28	−.58	—				
6. Emotional problems age 13 + 15 years	.12	−.19	−.14	−.32	−.14	—			
7. Memory age 43 years	.27	.48	.49	.35	−.20	−.13	—		
8. Memory age 53 years	.29	.49	.49	.35	−.19	−.13	.74	—	
9. Memory age 60+ years	.38	.47	.47	.33	−.20	−.08	.76	.75	—
*Note*. All coefficients were statistically significant at *p* < .01 except that between childhood cognition and sex (ns). Females were coded as 1 and males were coded as 0. Correlations between sex and other variables were based on biserial correlation. Model fit indices for the CFA model were χ^2^(73) = 143.074, root-mean-square error of approximation = .017, comparative fit index = .994, Tucker-Lewis index = .99.									
^a^ coded as higher score = better organization.									

**Table 4 tbl4:** Model Results of the SEM Path Diagram

Part A: Measurement model (factor loadings)						Part B: Structural model (path coefficients)					
	Estimate	95% CI		*SE*	*p*		Estimate	95% CI		*SE*	*p*
Cognition 43						Intercept (memory at 43)					
Item 1–43	1.00	na^a^	na^a^	na^a^	na^a^	Sex (0 = male, 1 = female)	0.52	0.43	0.62	0.05	0.00
Item 2–43	1.47	1.43	1.51	0.02	0.00	Cognition 8	0.43	0.37	0.49	0.03	0.00
Item 3–43	1.46	1.41	1.50	0.02	0.00	Education 26	0.46	0.40	0.51	0.03	0.00
						Emotional 13 + 15	−0.04	−0.10	0.02	0.03	0.19
Cognition 53						Conduct 13 + 15	−0.01	−0.08	0.05	0.03	0.70
Item 1–53	1.00	na^a^	na^a^	na^a^	na^a^	Organization 13 + 15^c^	0.11	0.04	0.19	0.04	0.00
Item 2–53	1.47	1.43	1.51	0.02	0.00	Slope (memory decline)					
Item 3–53	1.46	1.41	1.50	0.02	0.00	Sex (0 = male, 1 = female)	0.00	−0.09	0.09	0.05	0.97
						Cognition 8	0.01	−0.05	0.07	0.03	0.67
Cognition 60+						Education 26	−0.01	−0.07	0.05	0.03	0.72
Item 1–60+	1.00	na^a^	na^a^	na^a^	na^a^	Emotional 13 + 15	−0.01	−0.07	0.05	0.03	0.64
	1.47	1.43	1.51	0.02	0.00	Conduct 13 + 15	0.02	−0.05	0.09	0.04	0.59
Item 3–60+	1.46	1.41	1.50	0.02	0.00	Organization 13 + 15^c^	0.04	−0.04	0.12	0.04	0.30
						Education by 26					
						Sex (0 = male, 1 = female)	−0.29	−0.35	−0.24	0.03	0.00
Effect of test type on cognition factor^b^						Cognition 8	0.48	0.45	0.51	0.02	0.00
Cognition 43						Emotional 13 + 15	0.03	−0.01	0.06	0.02	0.13
Test type	0.22	0.11	0.32	0.05	0.00	Conduct 13 + 15	−0.07	−0.11	−0.03	0.02	0.00
						Organization 13 + 15^c^	0.25	0.21	0.29	0.02	0.00
Effect of test type on cognition factor^b^											
Cognition 53											
Test type	−0.01	−0.11	0.09	0.05	0.89	Emotional 13 + 15					
						Sex (0 = male, 1 = female)	0.20	0.12	0.27	0.04	0.00
						Cognition 8	−0.19	−0.23	−0.15	0.02	0.00
Cognition 60+											
Test type	0.09	−0.02	0.20	0.06	0.10	Conduct 13 + 15					
						Sex (0 = male, 1 = female)	−0.20	−0.27	−0.12	0.04	0.00
						Cognition 8	−0.17	−0.21	−0.13	0.02	0.00
Effect of test type on cognition items^b^						Organization 13 + 15					
Item 1–43						Sex (0 = male, 1 = female)	0.26	0.19	0.33	0.04	0.00
Test type	−0.15	−0.25	−0.04	0.05	0.01	Cognition 8	0.36	0.33	0.40	0.02	0.00
						Cognition 8					
						Sex (0 = male, 1 = female)	0.01	−0.06	0.08	0.04	0.85
Item 3–43						Covariance between intercept and slope					
Test type	0.19	0.08	0.30	0.06	0.00	intercept – slope	−0.03	−0.10	0.04	0.04	0.46
Item 1–53						Mean of slope (cognitive decline)					
Test type	0.28	0.17	0.39	0.06	0.00	Slope *M*^b^	−0.10	−0.16	−0.03	0.03	0.00
Item 1–60+						Residual variance of the slope (cognitive decline)					
Test type	−0.38	−0.50	−0.26	0.06	0.00	Slope residual variance	0.18	−0.32	0.68	0.26	0.48
^a^ The factor loading of first item was set to be 1 as reference indicator, therefore inferential statistics were not available. ^b^ We contrast coded the test type variable so that the mean of intercept and slope in the grow curve model represent the mean in the case that sex did not have a statistically significant effect on the slope. ^c^ coded as higher score = better organization.											

**Table 5 tbl5:** Indirect (Via Education) and Total Effects of Adolescent Mental Health on Baseline Memory and Rate of Memory Decline

	Indirect effects					Total effects				
Education as mediator	Estimate	95% CI		*SE*	*p*	Estimate	95% CI		*SE*	*p*
Mediation effect for intercept of cognitive aging										
Organization^a^	0.11	0.09	0.14	0.01	.00	0.23	0.15	0.30	0.04	.00
Emotional	0.01	0.00	0.03	0.01	.13	−0.03	−0.09	0.03	0.03	.38
Conduct	−0.03	−0.05	−0.01	0.01	.00	−0.05	−0.11	0.03	0.04	.21
Organization^a^	0.00	−0.02	0.01	0.01	.72	0.04	−0.04	0.11	0.04	.33
Emotional	0.00	0.00	0.00	0.00	.73	−0.01	−0.07	0.05	0.03	.64
Conduct	0.00	0.00	0.01	0.00	.72	0.02	−0.05	0.09	0.04	.58
CI = confidence interval. All effects were controlled for sex and childhood cognition; Direct effects are presented in Table 4, Part B.										
^a^ coded as higher score = better organization.
